# Supplementary material for: Hepatopancreas Proteomic Analysis Reveals Key Proteins and Pathways in Regulatory of Ovary Maturation of Macrobrachium nipponense
Source: Animals (Basel). 2023 Mar 8;13(6):977. doi: 10.3390/ani13060977 (PMC10044353; doi:10.3390/ani13060977)
Supplement: Supplementary file 1 [file animals-13-00977-s001.zip › Table S1.pdf]

Table S1 Primer used in this study.

Abbreviate name	Protein annotated name	Prime Forward	Prime Reverse
Af	Arylsulfatase	TACCGATCATTCCACACACAAGT	TCCAAACTGGAAAAAGAGGGTCA
FABP	Fatty acid-binding protein	TCACGGACACAGAGATGATTATGA	ATTGAACGTCGCATAAAGACCGA
Aff	Alpha-(l,6)-fucosyltransferase	TGACATCAGACAAGTCCTCCAAG	GTGTGCTGTATAGTCTCCGTAGG
Nct-1	NPC intracellular cholesterol transporter 1	ATCCCATTTCTGGTGTTAGCAGT	CAACCCGGCATATAGAGCAAATG
Caf	Choline O-acetyltransferase	GGCTAAGTGTCGAGAGGAATTGA	CATCAATGTTGGCTGGTTTGCTA
Shf	Serine hydroxymethyltransferase	TCAAGCTACTTCCACCTCCTCTA	GAAGGGTTTCTGAGTCTTCGCTA
So	Sarcosine oxidase	TTGAGATTAAAGGGACACGAGGG	CCCTTGTCGTAGTAACCCAGAAA
Smt	Sodium-dependent multivitamin transporter	TTTGAGTTCCTATTCGTCGCAGA	GAACCGATAGTGACCCAGAAGTT
Plgg-1	Protein lgg-l	TGAAGTGGCAATACAAGGAGGAG	CTTCATGGTGTTCCTGGTAGAGT
Gpd	Glyceraldehyde-3-phosphate dehydrogenase	GAAGTTCTCTCTCGACACCTCAC	GCTTCATTTCGTTGAAGACCTGG
Vg	Vitellogenin	GAAGTTAGCGGAGATCTGAGGT	CCTCGTTGACCAATCTTGAGAG
Gs	Glutamine synthetase	CAATGGATGGAGCCAGATACAGA	CGTTGAGTTCCTCCCTTGTTTTC
Cys -1	Cystatin-l	CCAAGAGAAGAGCGTGAAGTTTG	CATCCCCTCGTAGATTGTACTGG
Crp	Crustapin	CGTGTCATCCTTGGAAAGTTTC	ATTATTGTCCATGATGTACTGA
Llp	Legumain-like protein	GTTTTATGTAACGACCTCGGCTG	GTGTGATTGGTAATTGCACTCGT
EIF	Eukaryotic translation initiation factor	CATGGATGTACCTGTGGTGAAAC	CTGTCAGCAGAAGGTCCTCATTA
